# Real-World Treatment Patterns and Survival in Patients with *ROS1*-Positive Advanced Non-Small Cell Lung Cancer in Canada and Europe

**DOI:** 10.3390/curroncol33030152

**Published:** 2026-03-06

**Authors:** Winson Y. Cheung, Adam Lee, Helena Bote de Cabo, Kathrin Burdenski, Petros Christopoulos, Bárbara Pinto-Correia, Simon Deshayes, Nicolas Girard, Pooja Hindocha, Áine Madden, Marta Mella, Joana Moreira, Silvia Rizzi, Delvys Rodríguez Abreu, Marta Soares, Joseph Thomas, Maria Han, Christophe Y. Calvet, Gabrielle Emanuel, Mrudula B. Glassberg, Hazel Jacobs, Caroline Rault, Yong Yuan, Christos Chouaid

**Affiliations:** 1 Oncology Outcomes, University of Calgary, Calgary, AB T2N 1N4, Canada; 2 Health Economics & Outcomes Research, Oncology, Bristol Myers Squibb, Uxbridge UB8 1DH, UK; afc_adam14@hotmail.com; 3 Hospital Universitario 12 de Octubre, 28041 Madrid, Spain; helenabdc@usal.es; 4 University Cancer Center Frankfurt (UCT), 60590 Frankfurt am Main, Germany; burdenski@med.uni-frankfurt.de; 5 Thoraxklinik at Heidelberg University Hospital and Translational Lung Research Center Member of the German Center for Lung Research, 69126 Heidelberg, Germany; petros.christopoulos@med.uni-heidelberg.de; 6 IQVIA Ltd., Porto Salvo, 2740-266 Oeiras, Portugal; barbara.correia@iqvia.com (B.P.-C.); joanafilipa.canelasmoreira@iqvia.com (J.M.); 7 Center Hospitalier Universitaire de Caen, 14000 Caen, France; deshayes-si@chu-caen.fr; 8 Institut du Thorax Curie-Montsouris, Institut Curie, 75248 Paris, France; nicolas.girard2@curie.fr; 9 IQVIA Ltd., London W2 1AF, UK; pooja.hindocha@iqvia.com (P.H.); aine.madden@iqvia.com (Á.M.); joseph.thomas@iqvia.com (J.T.); 10 IQVIA Ltd., 20124 Milan, Italy; marta.mella@iqvia.com; 11 IQVIA Ltd., 2200 Copenhagen, Denmark; silvia.rizzi@iqvia.com; 12 Hospital Universitario Insular de Gran Canaria, 35016 Las Palmas de Gran Canaria, Spain; drodabr@gobiernodecanarias.org; 13 Portuguese Oncology Institute of Porto (IPO-Porto), 4200-162 Porto, Portugal; martasoares@ipoporto.min-saude.pt; 14 Vivantes Klinikum in Friedrichshain, 10249 Berlin, Germany; maria.han@vivantes.de; 15 Global Medical, Bristol Myers Squibb, Princeton, NJ 08540, USA; christophe.calvet@bms.com; 16 Real World Data Analytics, Bristol Myers Squibb, Uxbridge UB8 1DH, UK; gabrielle.m.emanuel@gmail.com; 17 Health Economics & Outcomes Research, Bristol Myers Squibb, Madison, NJ 07940, USA; mrudula.glassberg@bms.com; 18 Syneos Health, Farnborough GU14 7BF, UK; hazel.jacobs@bms.com; 19 Data Gnosis, 35000 Rennes, France; caroline.rault@bms.com; 20 Health Economics & Outcomes Research, Bristol Myers Squibb, Princeton, NJ 08540, USA; yongyuan.nj@gmail.com; 21 Centre Hospitalier Intercommunal de Créteil, 40 Avenue de Verdun, 94000 Créteil, France; christos.chouaid@chicreteil.fr

**Keywords:** non-small cell lung cancer, locally advanced, metastatic, *ROS1*, treatment patterns, survival, real world

## Abstract

Real-world data on patients with *ROS1*-positive advanced non-small cell lung cancer (NSCLC) remain scarce. In this descriptive observational retrospective cohort study, we describe characteristics, treatments, and real-world progression-free survival (rwPFS) and overall survival (OS) among patients with *ROS1*-positive advanced NSCLC (de novo or recurrent) using secondary data pooled from clinical sites in Canada, France, Germany, Portugal, and Spain as part of the Oncology Evidence Network. Site-specific patient inclusion periods occurred between 2009 and 2023, with follow-up to 2024, allowing ≥1 year of potential follow-up at each site. In total, 108 patients were included, with most (*n* = 105; 97.2%) having a de novo diagnosis of advanced NSCLC. 103 patients (95.4%) received ≥1 line of systemic anticancer therapy (SACT), of which 65 (63.1%) received first-line targeted therapy, mostly crizotinib monotherapy (*n* = 45) or crizotinib-based regimens (*n* = 10), with a median (95% CI) rwPFS and OS of 14.0 (8.3–19.8) and 47.9 (27.3–not estimable) months, respectively. Thirty-eight of the 103 SACT-treated patients (36.9%) received first-line non-targeted therapy, mostly platinum-based chemotherapy (*n* = 26); median (95% CI) rwPFS and OS were 9.0 (7.5–11.0) and 29.3 (17.7–65.7) months, respectively. Results from this study indicated a tendency for longer survival using currently available ROS1-targeted versus non-targeted therapy for patients with *ROS1*-positive advanced NSCLC. Nevertheless, survival outcomes were limited, highlighting the importance of more effective emerging treatments for *ROS1*-positive disease.

## Simple Summary

There is currently an insufficient amount of real-world information on treatments and outcomes among people with advanced-stage non-small cell lung cancer (NSCLC) who have mutations in the *ROS1* gene, termed *ROS1*-positive advanced NSCLC. We used pooled data collected between 2009 and 2024 from clinical sites in Canada, France, Germany, Portugal, and Spain to assess patients with *ROS1*-positive advanced NSCLC with the aim of describing patient characteristics, treatment patterns, and survival. In this study, results from 103 patients with *ROS1*-positive advanced NSCLC who received systemic anticancer therapy indicated a tendency for longer survival using currently available treatment that specifically targets *ROS1*-positive disease than with non-targeted treatments, such as chemotherapy. However, the observed survival outcomes were less than optimal, highlighting the importance of more effective emerging treatments for patients with *ROS1*-positive advanced NSCLC.

## 1. Introduction

About 50% of patients with non-squamous non-small cell lung cancer (NSCLC) carry actionable genomic alterations, including those in the *EGFR*, *KRAS*, *ALK*, or *ROS1* genes [1]. Due to the prevalence of these actionable alterations, North American and European guidelines recommend broad-spectrum biomarker testing for all patients with advanced non-squamous cell carcinoma and for certain subpopulations with squamous cell carcinoma (e.g., younger patients and never-smokers) [2,3,4]. Alterations in the *ROS1* gene, termed *ROS1* rearrangements, occur in approximately 0.5–3% of tested patients with NSCLC [5,6,7,8,9,10], and are more frequent in younger patients, female patients, never-smokers, and those with advanced disease; they are also more prevalent in lung adenocarcinomas than in other NSCLC histologies [5,6].

Crizotinib, a tyrosine kinase inhibitor (TKI) originally used to treat ALK-positive NSCLC, was the first agent to receive national/regional approvals in North America and Europe specifically for the treatment of patients with *ROS1*-positive disease, with approval in the United States and Europe in 2016 and in Canada in 2017 [11,12,13]. Entrectinib subsequently received national/regional approval for the treatment of *ROS1*-positive NSCLC in the United States in 2019 and in Canada and Europe in 2020 [14,15,16], with national/regional approval of repotrectinib following in the United States in 2023 and Canada and Europe in 2025 [17,18,19]. Recently, taletrectinib was also approved in the United States [20]. In alignment with these approvals, North American and European guidelines for patients with *ROS1*-positive advanced NSCLC were updated to recommend first-line treatment with crizotinib, entrectinib, or repotrectinib [3,4,21], with the latest North American guidelines also recommending taletrectinib [4]. Recommended second- and later-line approaches vary but typically involve use of an alternative ROS1-targeting TKI, an ALK-targeting TKI that has shown benefits in patients with *ROS1*-positive NSCLC (e.g., lorlatinib [22]), or, if no alternative TKI options are available, other systemic therapy approaches, such as platinum-based chemotherapy [3,4,21]. However, despite regional-level approvals and guideline-based recommendations, timings and decisions regarding reimbursement of ROS1-targeting therapies vary considerably across countries. For example, although crizotinib was approved for *ROS1*-positive disease in Europe in 2016, it has only been reimbursed in France since 2020 (with funding restricted to second- or later-line settings only) [23] and in Portugal since 2022 [24]. Likewise, despite the European approval of entrectinib in 2020, an unfavorable decision on reimbursement was recorded in France in 2021 [25], and it has only been reimbursed in Portugal since 2023 [26]. Similarly, although Canadian authorities approved crizotinib in 2017 and entrectinib in 2020, pan-Canadian reimbursement of these agents was not recommended until 2019 and 2021, respectively [27,28], with initiation of provincial reimbursement occurring even later (e.g., funding of crizotinib in the province of Alberta was only initiated in July 2020) [29].

Real-world data on patients with *ROS1*-positive advanced NSCLC remain scarce, for both those treated with ROS1-targeting therapies and those not receiving targeted treatments. Moreover, based on real-world studies showing a median overall survival (OS) of only 2–3 years after first- or second-line crizotinib [30,31,32], and clinical studies suggesting comparable efficacy for crizotinib and entrectinib [33,34], there remains an unmet need for alternative therapies for *ROS1*-positive disease. As such, further real-world studies could provide useful insights into clinical care pathways, treatment decisions, and survival outcomes in this patient population. Onco-Optimise (formerly known as I-O Optimise) is an ongoing, international collaborative research initiative designed to provide timely insights into the evolving management of various thoracic malignancies, including NSCLC [35,36]. As part of Onco-Optimise, the present study was designed to leverage data from multiple high-quality real-world data sources to describe demographic and clinical characteristics, treatment patterns, and associated survival outcomes among patients with *ROS1*-positive advanced NSCLC in Canada and Europe.

## 2. Materials and Methods

### 2.1. Study Design and Patient Population

This descriptive observational retrospective cohort study was conducted using secondary data pooled from clinical sites included in the Oncology Evidence Network (OEN). The OEN is a collaboration of large hospital centers with strong clinical informatics capabilities across multiple countries (primarily Canada and European countries) that work with the support of an industrial partner (IQVIA) to provide high-quality real-world oncology data reflecting routine clinical care [37]. For this study, data were derived from patient electronic medical records collected at Oncology Outcomes in Canada (using data from the province of Alberta); the Institut Curie and Center Hospitalier Universitaire de Caen in France; the University Cancer Center Frankfurt, Thoraxklinik at Heidelberg University Hospital, and Vivantes Klinikum in Friedrichshain in Germany; the Portuguese Oncology Institute of Porto in Portugal; and the Hospital Universitario Insular de Gran Canaria and Hospital Universitario 12 de Octubre in Spain. Based on the design of the OEN, a common data model was implemented across these sites to ensure data harmonization and consistency in data curation and reporting [37].

The site-specific patient inclusion periods for this study all occurred between 2009 and 2023 and were driven by the respective timings of *ROS1* rearrangement testing implementation at each site. Patients were included in the study at their date of diagnosis of advanced NSCLC (inclusion date) and followed until death or end of the study period, whichever occurred first. Site-specific follow-up periods extended from the start date of each site-specific patient inclusion period up to 2024, allowing at least 1 year of potential follow-up at each contributing site before the end of the study period.

The study population included all patients within the participating sites who were aged at least 18 years at initial lung cancer diagnosis and who had advanced NSCLC (including patients with a de novo diagnosis and those with recurrent disease) and confirmed *ROS1*-positive disease. A “de novo diagnosis” refers to patients with newly diagnosed locally advanced or metastatic (stage III or IV) NSCLC whose initial treatment was non-curative and who received a first line of systemic anticancer therapy (SACT) or best supportive care (BSC). “Recurrent disease” refers to patients first diagnosed with stage I–III NSCLC who received curative treatment (protocol-defined as surgery, radiotherapy, neoadjuvant therapy, and/or adjuvant therapy administered in the curative setting) but whose disease progressed to advanced NSCLC before receiving a first line of SACT or BSC, with the date of recurrence captured at the participating sites. Confirmation of *ROS1*-positive status was based on tests performed between 30 days before diagnosis of advanced NSCLC and the end of the site-specific patient inclusion period. Patients required a positive *ROS1* test using a non-immunohistochemistry technique (e.g., fluorescence in situ hybridization or next-generation sequencing) or a positive *ROS1* test using immunohistochemistry, but without evidence of a negative test using non-immunohistochemistry technique. Patients were excluded if they had any previous primary malignancy (excluding non-melanoma skin cancer) in the 5 years before initial NSCLC diagnosis or at any point after their diagnosis date, or if they participated in a clinical trial before the start of their first line of SACT.

Analyses were conducted for the overall population and stratified by Canadian versus European sites.

### 2.2. Variables

Data on patient demographic and clinical characteristics were collected at the time of initial NSCLC diagnosis and/or diagnosis of advanced NSCLC. Testing patterns for *ROS1* rearrangements were assessed in the full study population. For the classification of initial treatment, patients were categorized as receiving either SACT (patients who were administered at least one line of SACT within the first 180 days after diagnosis of advanced NSCLC) or BSC (patients who were either administered a treatment for management of disease-related symptoms only or were untreated within the first 180 consecutive days after diagnosis of advanced NSCLC). For this analysis, SACT was further categorized as “platinum-based chemotherapy alone,” “non-platinum-based chemotherapy alone,” “targeted therapy,” “anti-programmed death-(ligand) 1 [PD-(L)1] immune checkpoint inhibitor (ICI) monotherapy,” “anti-PD-(L)1 ICI plus chemotherapy,” or “other monoclonal antibodies,” as defined in Supplementary Table S1. Lines of therapy and treatment sequences were determined by each participating site based on available data.

Real-world progression-free survival (rwPFS) and OS were assessed in patients receiving SACT. For this analysis, rwPFS was defined as the time from the start date of first line of SACT to the date of recorded disease progression or date of death from any cause, with the date of recorded disease progression based on radiological and/or clinical progression as assessed by physicians at the respective clinical sites. Patients without a recorded rwPFS event were censored at the earliest of the start of second line of therapy, the date of loss to follow-up, the date of starting a clinical trial, or the end of the study period. OS was defined as the time from start date of first line of SACT to the date of death due to any cause. Patients without an OS event were censored at the earliest of the date of loss to follow-up, the date of starting a clinical trial, or the end of the study period.

### 2.3. Statistical Analysis

Descriptive statistics were used for data on demographic and clinical characteristics, *ROS1* rearrangement testing patterns, and treatment patterns. Time-to-event endpoints (rwPFS and OS) were described using the Kaplan–Meier method. For subgroups with fewer than 20 patients, both the Kaplan–Meier curve and associated median value were suppressed; additionally, all Kaplan–Meier curves were truncated when the number of patients at risk fell below 10. Based on OEN study requirements regarding the privacy of Canadian data, for any data stratified by geographic region (Canada vs. Europe), patient numbers between one and nine were masked and patient numbers ≥10 were rounded to the nearest 10, with associated percentages rounded to the nearest 5%. No imputation methods were used to handle missing data except for date variables. Due to the descriptive nature of the study, no statistical testing was performed and confounding adjustment was not conducted.

## 3. Results

### 3.1. Patients

In total, 108 patients with confirmed *ROS1*-positive advanced NSCLC were included (Table 1). Overall, the median (interquartile range [IQR]) duration of follow-up from diagnosis of advanced NSCLC was 24.1 (10.9–46.8) months and was similar among patients from Canada (26.5 [11.2–43.4] months) and Europe (23.4 [10.9–44.4] months). One hundred and five patients (97.2%) had a de novo diagnosis of advanced NSCLC, with 89 of these patients (82.4% of the overall study population) having metastatic (stage IV) disease at diagnosis and the remaining 16 (14.8% of the overall study population) having locally advanced disease at diagnosis (stage IIIA, *n* = 4; IIIB, *n* = 9; IIIC, *n* = 3). Only three patients (2.8%) had recurrent advanced disease, after initial diagnosis at stage IB (*n* = 1), IIIA (*n* = 1) or IIIC (*n* = 1). For these three patients, the median (IQR) time from initial diagnosis of NSCLC to advanced diagnosis was 24.7 (4.6–24.7) months.

Overall, median (IQR) age at diagnosis of advanced NSCLC was 57 (48–68) years, 68 patients (63.0%) were female, 106 (98.1%) had non-squamous cell NSCLC (all adenocarcinoma), 58 (53.7%) were never-smokers, and 20 (18.5%) had brain metastases at diagnosis of advanced NSCLC (Table 1). Ten patients (9.3%) were diagnosed with advanced NSCLC between 2009 and 2015 (i.e., before first formal approval of crizotinib for *ROS1*-positive disease), a further 57 (52.8%) were diagnosed with advanced NSCLC between 2016 and 2019 (i.e., after first regional/country-specific approval of crizotinib for *ROS1*-positive disease, but before reimbursement in this setting in most of the participating countries), and the remaining 41 patients (38.0%) were diagnosed between 2020 and 2023. Characteristics of the patients from Canada and Europe were mostly similar, although the Canadian population was slightly younger and included a larger proportion of never-smokers (Supplementary Table S2).

### 3.2. ROS1 Testing

Most testing samples originated from tissue (*n* = 95; 88.0%) and came from the primary tumor site (*n* = 60; 55.6%; Table 2). Overall, 78 patients (72.2%) underwent multi-gene (gene panel) testing, with the most common techniques being next-generation sequencing (*n* = 48; 44.4%) or fluorescence in situ hybridization (*n* = 45; 41.7%). The majority of patients (*n* = 98; 90.7%) were tested for *ROS1* rearrangements after diagnosis of advanced NSCLC, with a median (IQR) time from diagnosis to testing of 31.0 (11.0–110.0) days; 10 patients (9.3%) were tested for *ROS1* rearrangements before diagnosis of advanced NSCLC, with a median (IQR) time from testing to diagnosis of 6.0 (1.0–9.0) days (Table 2).

### 3.3. Treatment Patterns and Pathways

Of the 108 included patients, 103 (95.4%) received at least one line of SACT and five (4.6%) received BSC. Sixty-five of the 103 patients treated with SACT (63.1%) received first-line targeted therapy, most commonly crizotinib monotherapy (*n* = 45; 43.7%), crizotinib-based combination regimens (*n* = 10; 9.7%), or entrectinib monotherapy (*n* = 9; 8.7%) (Table 3). Thirty-eight of the 103 patients treated with SACT (36.9%) received first-line non-targeted therapy, most commonly platinum-based chemotherapy (*n* = 26; 25.2%) (Table 3). First-line use of anti-PD-(L)1 ICIs plus chemotherapy, anti-PD-(L)1 ICI monotherapy, and non-platinum chemotherapy was limited (Table 3).

**Figure 1:**
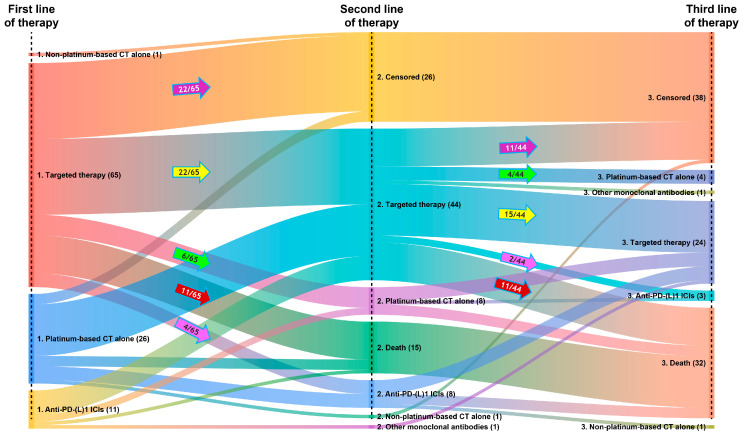
Treatment sequences (first to third line of therapy) for all patients receiving at least one line of SACT (*N* = 103). The visual thickness of the horizontal lines reflects the number of patients following a specific treatment sequence across the first, second, and third line of therapy. In addition, the colored arrows provide specific data on treatment sequences for patients receiving targeted therapy at first or second line of therapy, as follows: purple arrows, targeted therapy followed by censorship; yellow arrows, targeted therapy followed by targeted therapy; green arrows, targeted therapy followed by platinum-based CT alone; red arrows, targeted therapy followed by death; and pink arrows, targeted therapy followed by anti-PD-(L)1 ICIs. Abbreviations: CT, chemotherapy; ICI, immune checkpoint inhibitor; PD-(L)1, programmed death-(ligand) 1.

Of the 103 patients treated with SACT, 15 (14.6%) died and 26 (25.2%) were censored during first-line treatment; 62 patients (60.2%) went on to receive a second line of therapy (Figure 1). Forty-four of the 62 patients (71.0%) treated with a second line of SACT received second-line targeted therapy, most commonly crizotinib monotherapy (*n* = 19; 30.6%) or lorlatinib monotherapy (*n* = 12; 19.4%) (Table 3). Eighteen of the 62 patients (29.0%) treated with a second line of therapy received second-line non-targeted therapy, most commonly platinum-based chemotherapy (*n* = 8; 12.9%) or anti-PD-(L)1 ICI monotherapy (*n* = 7; 11.3%) (Table 3).

Of the 62 patients who received a second line of SACT, 17 (27.4%) died and 12 (19.4%) were censored during second-line treatment; 33 patients (53.2%) went on to receive a third line of therapy (Figure 1). Twenty-four of the 33 patients treated with a third line of SACT (72.7%) received third-line targeted therapy, most commonly lorlatinib monotherapy (*n* = 15; 45.5%) (Table 3). Only 17 of the 103 patients administered at least one line of SACT (16.5%) went on to receive a fourth line of therapy, and only five (4.9%) received a fifth line of therapy.

The most common treatment sequences from first to second line of therapy involved targeted therapies followed by targeted therapies. During first-line treatment, 33 of the 65 patients who received first-line targeted therapy died or were censored; the remaining 32 patients went on to receive second-line therapy, of whom 22 (68.8%) were treated with a second line of targeted therapy (Figure 1). Similarly, the most common treatment sequences from second to third line of therapy also involved targeted therapies followed by targeted therapies. During second-line treatment, 22 of the 44 patients who received second-line targeted therapy died or were censored; the remaining 22 patients went on to receive a third line of therapy, of whom 15 (68.2%) were treated with third-line targeted therapy (Figure 1).

### 3.4. Survival Outcomes

**Figure 2:**
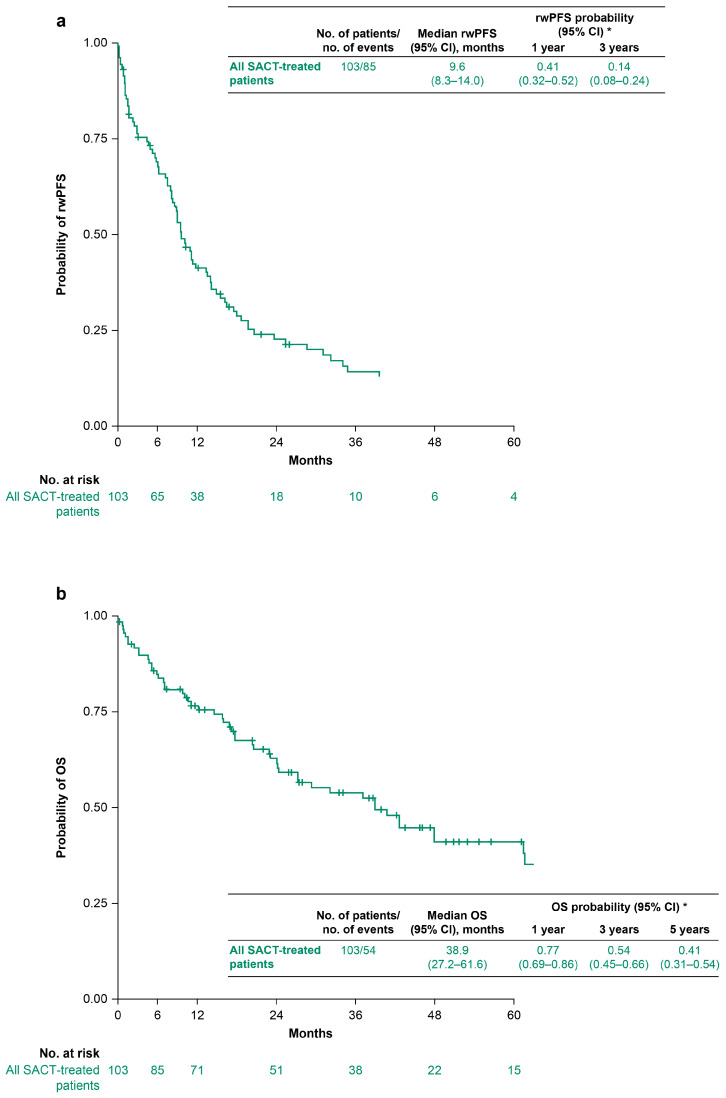
rwPFS (**a**) and OS (**b**) for all patients receiving at least one line of SACT (*N* = 103). Vertical tick marks indicate censored data. * Survival probabilities are suppressed when the number of patients at risk is <10. Abbreviations: CI, confidence interval; OS, overall survival; rwPFS, real-world progression-free survival.

**Figure 3:**
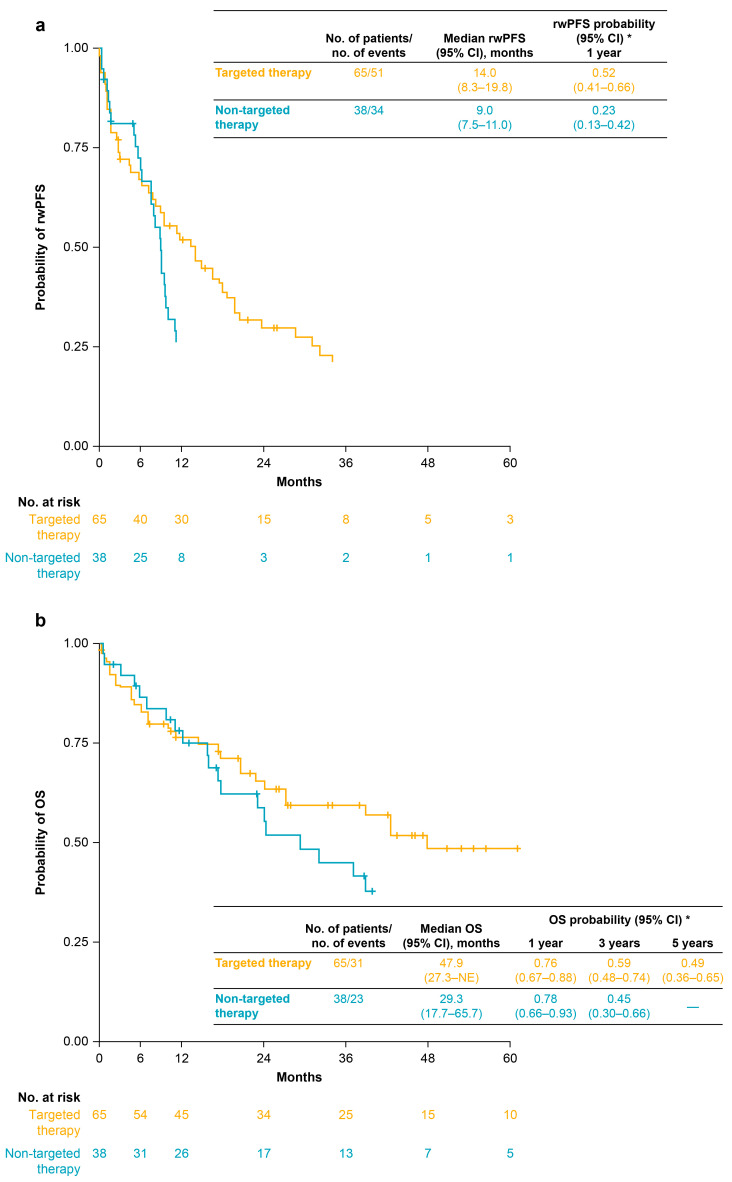
rwPFS (**a**) and OS (**b**) for patients receiving first-line targeted therapy (*n* = 65) and patients receiving first-line non-targeted therapy (*n* = 38). Targeted therapy included TKI monotherapies or combinations of TKIs with other classes of treatment; non-targeted therapy included platinum-based chemotherapy alone, non-platinum-based chemotherapy alone, anti-PD-(L)1 ICI monotherapy, or combinations of anti-PD-(L)1 ICIs with chemotherapy (see Table 3). Vertical tick marks indicate censored data. * Survival probabilities are suppressed when the number of patients at risk is <10. Abbreviations: CI, confidence interval; ICI, immune checkpoint inhibitor; NE, not estimable; OS, overall survival; PD-(L)1, programmed death-(ligand) 1; rwPFS, real-world progression-free survival; TKI, tyrosine kinase inhibitor.

Among the 103 patients treated with SACT, median (95% confidence interval [CI]) rwPFS was 9.6 (8.3–14.0) months and median (95% CI) OS was 38.9 (27.2–61.6) months (Figure 2). For the 65 patients who received first-line targeted therapy (i.e., mostly crizotinib monotherapy or crizotinib-based combination regimens), median (95% CI) rwPFS and OS were 14.0 (8.3–19.8) months and 47.9 (27.3–not estimable) months, respectively (Figure 3). For the 38 patients who received first-line non-targeted therapy (i.e., platinum-based chemotherapy alone, non-platinum-based chemotherapy alone, anti-PD-[L]1 ICI monotherapy, or combinations of anti-PD-[L]1 ICIs with chemotherapy), median (95% CI) rwPFS and OS were 9.0 (7.5–11.0) months and 29.3 (17.7–65.7) months, respectively (Figure 3).

In SACT-treated patients from Canada, median (95% CI) rwPFS and OS were 8.3 (4.4–15.6) months and 42.6 (27.3—not estimable) months, respectively; in SACT-treated patients from Europe, they were 11.0 (8.9–16.5) months and 38.9 (23.1–65.7) months, respectively (Supplementary Figure S1).

## 4. Discussion

Using data retrospectively pooled from clinical sites in Canada and four European countries (France, Germany, Portugal, and Spain) between 2009 and 2024, this descriptive real-world observational study provides insights into patient demographic and clinical characteristics, testing patterns, treatment patterns and pathways, and survival outcomes for patients with advanced NSCLC carrying the relatively rare *ROS1* rearrangement.

The population of patients providing data for this study had a relatively low median age (vs. patients with NSCLC not carrying actionable genomic alterations) and contained a predominance of female patients, never-smokers, and patients with adenocarcinoma, which aligns with literature describing common characteristics of patients with *ROS1* rearrangements [5,6]. The study population was also similar to *ROS1*-positive patient populations in other real-world studies conducted in North America, Europe, and Asia [30,31,32,38,39,40,41,42].

The most commonly administered treatment types in this study were ROS1-targeted therapies, which were received by 63% of patients as first line of therapy, by 71% as second line of therapy, and by 73% as third line of therapy. The most common treatment was crizotinib (as monotherapy or in combination regimens) as first and second line of therapy, and lorlatinib monotherapy as third line of therapy. It was also noteworthy that the most common treatment sequences from first to second line of therapy and from second to third line of therapy involved targeted therapies followed by targeted therapies. The treatment patterns observed in this study were reflective of treatment approvals and availability in the participating regions and countries over the study period. With the analyses being conducted between 2009 and 2024, crizotinib would have been the only ROS1-targeted therapy approved in North America and Europe for most of the study [11,12,30]. Moreover, although entrectinib was approved for the treatment of *ROS1*-positive NSCLC in both Canada and Europe in 2020, it was restricted to patients who had not previously received crizotinib (in Canada) or who had not previously received any ROS1 inhibitor (in Europe) [15,16], which likely limited its use beyond the first-line setting in this study, and probably contributed to the high use of lorlatinib (an ALK inhibitor frequently used off label in patients with *ROS1*-positive NSCLC) in the second- and third-line settings. Finally, it is important to consider the influence of country-specific reimbursement decisions on the observed treatment patterns. As detailed in the Introduction, there was substantial variability in official timings and decisions regarding reimbursement of ROS1-targeting therapies across the countries participating in this study, a feature supported by a survey of pathologists or clinical scientists in molecular pathology and oncologists conducted across numerous European countries in 2023 [43]. Overall, despite Canadian and European regional-level approvals of crizotinib in 2016/2017 [12,13] and entrectinib in 2020 [15,16], both drugs were not reimbursed for the first-line treatment of patients with *ROS1*-positive NSCLC in most of the participating countries for most of the study period, which likely contributed to the proportion of patients in this study receiving first-line non-targeted therapy for *ROS1*-positive disease.

The median rwPFS and OS reported in this study for patients with *ROS1*-positive advanced NSCLC receiving first-line targeted therapy (mostly comprising crizotinib alone or in crizotinib-based combination regimens) was 14.0 and 47.9 months, respectively. These values align with ranges of survival reported in other real-world studies (PFS, 8.6–29.0 months; OS, 16.2–36.2 months) [30,31,32,38,42] and clinical trials (PFS, 15.9–22.8 months; OS, 32.5–54.8 months) [44,45,46,47,48] conducted across the world and where patients were treated with crizotinib. However, assessing outcomes from the current study in the context of other relevant real-world studies and clinical trials is difficult due to the relative scarcity of real-world data, as well as differences in study designs and patient populations. A particular challenge is that many of the previous studies have evaluated the efficacy of crizotinib when used as either first- or second-line therapy for advanced *ROS1*-positive NSCLC or even when used at any point in the treatment pathway [32]. The impact that this has on survival outcomes was demonstrated in a recent global meta-analysis of real-world studies of crizotinib for *ROS1*-positive advanced NSCLC, which showed that the pooled median PFS with crizotinib at any line of therapy was 14.5 months, whereas the pooled median PFS for first-line crizotinib was 18.1 months [49]. In addition, while many of the *ROS1*-positive patient populations have similar overarching characteristics (e.g., a high proportion of female patients and never-smokers), they vary in other characteristics that might impact survival. For example, clinical trials of crizotinib typically included no or only a small proportion of patients with Eastern Cooperative Oncology Group performance status >1 [44,45,46,47], whereas several of the real-world study populations included more than 10% of such patients [30,31,42], which has been shown to impact OS outcomes [31]. It is also noteworthy that crizotinib has poor intracranial penetration, and patients with existing brain metastases have shown worse survival outcomes versus those without in both clinical trials and real-world analyses [30,32,44,47]. As such, the relative proportions of patients with brain metastases (both present at start of therapy and developing during treatment) would likely impact survival outcomes in the respective studies/trials.

Results from this study indicated a tendency for longer survival with first-line ROS1-targeted therapy (mostly crizotinib) than with first-line non-targeted therapy. Although this appears to further support the benefits of crizotinib as first-line treatment for *ROS1*-positive advanced NSCLC, the observed median rwPFS of 14.0 months and OS of 47.9 months (i.e., approximately 1 and 4 years, respectively) from the start of treatment remain limited, especially considering the relatively young age of the *ROS1*-positive population (median age of 57 years). Despite these useful insights, it is important to acknowledge potential confounding factors for the comparison of patients receiving targeted versus non-targeted therapy in this study, including possible differences in baseline characteristics (e.g., Eastern Cooperative Oncology Group performance status, comorbidities, or metastatic sites) between these treatment subgroups. Additionally, the inclusion of patients diagnosed with advanced *ROS1*-positive NSCLC before availability/reimbursement of ROS1-targeted therapy (as well as anti-PD-[L]1 ICIs) in their respective countries (i.e., pre-2015/2016) may have impacted these outcomes. For these patients, chemotherapy would have been the only therapeutic option, regardless of whether they would be considered suitable for chemotherapy if diagnosed after more contemporary treatments became available.

Real-world data on first-line use of entrectinib for *ROS1*-positive advanced NSCLC are scarce, but results from an integrated analysis of three clinical trials (reporting a median PFS of 15.7 months and a median OS of 47.8 months) and from a simulated treatment comparison analysis based on phase 1–2 studies have suggested that entrectinib is unlikely to provide significant survival improvements over crizotinib [33,34]. Similarly, real-world data on the first-line use of repotrectinib are limited due to its recent approval. However, in the TRIDENT-1 trial of repotrectinib for patients with TKI-naive *ROS1*-positive advanced NSCLC, median PFS was 35.7 months, median OS was not reached (with an 18-month OS rate of 88%), and encouraging clinical activity against intracranial disease was observed [50]. Moreover, in a recent indirect comparison analysis in patients with TKI-naïve *ROS1*-positive locally advanced or metastatic NSCLC, repotrectinib was associated with statistically significant improvements in PFS, and numerically improved objective response rate and duration of response relative to crizotinib and entrectinib [51]. In addition, although real-world data are unavailable at present, two other novel ROS1-targeting therapies, taletrectinib and zidesamtinib, have shown encouraging efficacy and safety data in patients with *ROS1*-positive NSCLC [52]. Taken together, these emerging data suggest the potential for further improvements in survival outcomes for this patient population.

Several limitations should be considered when interpreting the data presented here. First, as is typical for real-world analyses of rare diseases, the study included a relatively small overall sample size, which could impact data interpretation, particularly for some of the smaller subgroups assessed. Second, the study used secondary data from electronic medical records, which could have been subject to point-of-care data entry errors (information bias or measurement errors) that would not be detected nor corrected during the analyses. Third, due to the descriptive nature of the study, there was no statistical testing performed and no adjustments for potential confounding factors. Fourth, due to masking rules preventing a comprehensive review of Canadian patients, and because data for individual European countries were not available, the study was not able to determine if there were any country-specific differences that might have influenced the results. Fifth, possible country- or site-specific differences in the measurement and/or recording of disease progression could have impacted the reported rwPFS outcomes. Sixth, and finally, the study collected data on treatment patterns and/or survival outcomes between 2020 and 2022, and these data may have been impacted by the COVID-19 pandemic. Despite these limitations, a strength of the current study was the use of high-quality, real-world data sources that helped provide a holistic view of the treatment landscape for patients with *ROS1*-positive advanced NSCLC across Canada and Europe. Furthermore, the use of multiple data sources across the various countries ensured that sufficient data were available for the analysis of patients with the relatively rare *ROS1* rearrangement, with the included sample (*N* = 108) larger than many previous real-world studies of *ROS1*-positive NSCLC [30,38,40,41]. Finally, the harmonization of data across sites using a common data model was important in ensuring a consistent approach to data curation and reporting throughout the current analyses [37].

## 5. Conclusions

This study provides unique multicountry insights into the demographic and clinical characteristics of patients with *ROS1*-positive advanced NSCLC and associated treatment patterns and survival outcomes. Although the results indicated a tendency for longer survival using currently available ROS1-targeted therapy versus non-targeted therapy for patients with *ROS1*-positive advanced NSCLC, outcomes were limited, highlighting the importance of more effective emerging therapeutic options for *ROS1*-positive disease.

## Figures and Tables

**Table 1:** Demographic and clinical characteristics for the full study population.

	All Patients (*N* = 108)
Age *^,†^	
Median (range), years	57 (28–89)
IQR	48–68
<75 years, *n* (%)	94 (87.0)
Sex, *n* (%) ^‡^	
Female	68 (63.0)
Male	40 (37.0)
Histology, *n* (%) ^‡^	
Non-squamous cell NSCLC	106 (98.1)
Adenocarcinoma	106 (98.1)
Squamous cell NSCLC	0
Other specified	1 (<1.0)
Missing	1 (<1.0)
Tumor stage, *n* (%) ^‡^	
I–II	1 (<1.0)
III (IIIA–IIIC)	18 (16.7)
IV	89 (82.4)
Year of advanced diagnosis, *n* (%) ^†^	
2009–2015	10 (9.3)
2016–2019	57 (52.8)
2020–2023	41 (38.0)
Type of advanced diagnosis, *n* (%) ^†,§^	
De novo diagnosis	105 (97.2)
Recurrent disease	3 (2.8)
ECOG PS, *n* (%) ^†^	
0–1	63 (58.3)
>1	9 (8.3)
Missing	36 (33.3)
Smoking status, *n* (%) ^†^	
Current smoker	17 (15.7)
Past smoker	29 (26.9)
Never-smoker	58 (53.7)
Missing/unknown	4 (3.7)
Presence of comorbidities, *n* (%) ^†,‖^	
Hypertension	13 (12.0)
Venous thromboembolism	8 (7.4)
Diabetes mellitus	8 (7.4)
Congestive heart failure	3 (2.8)
Presence of brain metastases, *n* (%)	
At diagnosis of advanced NSCLC	20 (18.5)
Before first line of therapy ^¶^	23 (21.3)
Total no. of brain, liver and/or bone metastases, *n* (%) ^†^	
0	59 (54.6)
1	33 (30.6)
2	14 (13.0)
≥3	2 (1.9)

* One patient had missing data on age. ^†^ Data recorded at diagnosis of advanced NSCLC. ^‡^ Data recorded at initial diagnosis of NSCLC. ^§^ “De novo diagnosis” refers to patients with newly diagnosed stage III or IV NSCLC whose initial treatment was non-curative and who received a first line of SACT or BSC; “recurrent disease” refers to patients first diagnosed with stage I–III NSCLC who received curative treatment but progressed to advanced disease before receiving a first line of SACT or BSC. ^‖^ No patients had chronic lower respiratory disease, arrhythmia, or liver disease at diagnosis of advanced NSCLC. ^¶^ Total of all patients with brain metastases at diagnosis of advanced NSCLC (*n* = 20) and patients with brain metastases detected between diagnosis and first line of SACT (*n* = 3). Abbreviations: BSC, best supportive care; ECOG PS, Eastern Cooperative Oncology Group performance status; IQR, interquartile range; NSCLC, non-small cell lung cancer; SACT, systemic anticancer therapy.

**Table 2:** Testing patterns for *ROS1* rearrangements among the full study population.

	All Patients (*N* = 108)
Sample type, *n* (%)	
Tissue	95 (88.0)
Blood	3 (2.8)
Other	6 (5.6)
Missing/unknown	4 (3.7)
Site of tissue collection, *n* (%)	
Primary tumor	60 (55.6)
Metastases	37 (34.3)
Missing/unknown	11 (10.2)
Type of test, *n* (%)	
Multi-gene (gene panel)	78 (72.2)
Single gene	27 (25.0)
Missing/unknown	3 (2.8)
Testing technique, *n* (%)	
Next-generation sequencing	48 (44.4)
Fluorescence in situ hybridization	45 (41.7)
Immunohistochemistry	13 (12.0)
Missing/unknown	2 (1.9)
Timing of test	
Before advanced NSCLC diagnosis, *n* (%)	10 (9.3)
Median time from test to diagnosis (IQR), days	6.0 (1.0–9.0)
After advanced NSCLC diagnosis	98 (90.7)
Median time from diagnosis to test (IQR), days	31.0 (11.0–110.0)

Abbreviations: IQR, interquartile range; NSCLC, non-small cell lung cancer.

**Table 3:** Treatment patterns for patients receiving SACT at first, second, and third line of therapy.

	First Line of Therapy (*n* = 103)	Second Line of Therapy (*n* = 62)	Third Line of Therapy (*n* = 33)
Targeted therapy, *n* (%)	65 (63.1)	44 (71.0)	24 (72.7)
Crizotinib monotherapy	45 (43.7)	19 (30.6)	4 (12.1)
Crizotinib in combination regimens *	10 (9.7)	2 (3.2)	0
Entrectinib monotherapy	9 (8.7)	3 (4.8)	1 (3.0)
Lorlatinib monotherapy	0	12 (19.4)	15 (45.5)
Other TKI-based regimens ^†^	1 (1.0)	8 (12.9)	4 (12.1)
Non-targeted therapy, *n* (%)	38 (36.9)	18 (29.0)	9 (27.3)
Platinum-based chemotherapy alone ^‡^	26 (25.2)	8 (12.9)	4 (12.1)
Anti-PD-(L)1 ICI plus chemotherapy	7 (6.8)	1 (1.6)	2 (6.1)
Anti-PD-(L)1 ICI monotherapy	4 (3.9)	7 (11.3)	1 (3.0)
Non-platinum-based chemotherapy alone	1 (1.0)	1 (1.6)	1 (3.0)
Other monoclonal antibodies	0	1 (1.6)	1 (3.0)

* For first line of therapy, four patients received crizotinib plus chemotherapy, two received crizotinib plus chemotherapy plus an anti-PD-(L)1 ICI, three received crizotinib plus an anti-PD-(L)1 ICI, and one received crizotinib plus a monoclonal antibody. For second line of therapy, one patient received crizotinib plus another TKI and one received crizotinib plus an anti-PD-(L)1 ICI. ^†^ For first line of therapy, one patient received monotherapy with a TKI other than crizotinib, entrectinib, or lorlatinib. For second line of therapy, six patients received monotherapy with TKIs other than crizotinib, entrectinib, or lorlatinib; one received a combination of two TKIs; and one received a TKI plus chemotherapy combination. For third line of therapy, three patients received monotherapy with a TKI other than crizotinib, entrectinib, or lorlatinib, and one received a TKI plus chemotherapy combination. ^‡^ Across the three lines of therapy, most patients received carboplatin plus pemetrexed (*n* = 20). Abbreviations: ICI, immune checkpoint inhibitor; PD-(L)1, programmed death-(ligand) 1; SACT, systemic anticancer therapy; TKI, tyrosine kinase inhibitor.

## Data Availability

Data presented in this article are not publicly available, and no data sharing is planned. Patient-level data cannot to be shared due to relevant regulatory and confidentiality reasons. Aggregate results are presented in this article.

## Supplementary Materials

The following supporting information can be downloaded at: https://www.mdpi.com/article/10.3390/curroncol33030152/s1, Table S1: Definition of SACT categories; Table S2: Select demographics and clinical characteristics for the full study population stratified by geographic region; Figure S1: rwPFS (a) and OS (b) for patients receiving at least one line of SACT stratified by geographic region.
