# Supplementary material for: Real-World Treatment Patterns and Survival in Patients with ROS1-Positive Advanced Non-Small Cell Lung Cancer in Canada and Europe
Source: Curr Oncol. 2026 Mar 6;33(3):152. doi: 10.3390/curroncol33030152 (PMC13025971; doi:10.3390/curroncol33030152)
Supplement: Supplementary file 1 [file curroncol-33-00152-s001.zip › curroncol-4078018-supplementary.pdf]

Supplementary data

Table S1. Definition of SACT categories.

Category	Definition
Platinum-based chemotherapy alone	Any platinum-based chemotherapy regimen (singlet, doublet, triplet, or combinations of ≥4 chemotherapy agents) but not combinations of platinum-based chemotherapy with other classes of treatment, e.g., TKIs, PD-(L)1 ICIs, or other monoclonal antibodies
Non-platinum-based chemotherapy alone	Any non-platinum-based chemotherapy regimen (singlet or doublet) but not combinations of non-platinum-based chemotherapy with other classes of treatment, e.g., TKIs, anti-PD-(L)1 ICIs, or other monoclonal antibodies
Targeted therapy	TKI monotherapies, combinations of TKIs with other TKIs, or combinations of TKIs with other classes of treatment, e.g., chemotherapy, anti-PD-(L)1 ICIs, or other monoclonal antibodies
Anti-PD-(L)1 ICI monotherapies	Any single-agent anti-PD-(L)1 ICI
Anti-PD-(L)1 ICI plus chemotherapy	Any combination of anti-PD-(L)1 ICIs with platinum- or non-platinum-based chemotherapy singlets, doublets, etc
Other monoclonal antibodies	Any single-agent monoclonal antibody that is not categorized as a TKI or an anti-PD-(L)1 ICI

Abbreviations: ICI, immune checkpoint inhibitor; PD-(L)1, programmed death-(ligand) 1; SACT, systemic anticancer therapy; TKI, tyrosine kinase inhibitor.

Table S2. Select demographics and clinical characteristics for the full study population stratified by geographic region. *

	**Canada (N = 40)**	**Europe (N = 70)**
Age †,‡		
Median, years	52.5	59
IQR	46–63	50–70
Female sex, n (%) §	30 (70)	40 (60)
Non-squamous cell histology, n (%) §	40 (100)	70 (100)
Stage IV disease, n (%) §	30 (80)	60 (85)
De novo advanced diagnosis, n (%) ‖	40 (100)	70 (95)
ECOG PS 0–1, n (%) ‡	20 (60)	40 (60)
Never-smoker, n (%) ‡	30 (70)	30 (45)
Presence of hypertension, n (%) ‡	0	10 (20)
Presence of brain metastases, n (%) ‡	PM	20 (25)
No. of brain, liver and/or bone metastases, n (%) ‡		
0	20 (65)	30 (50)
1	PM	20 (35)
2	PM	10 (15)
≥3	PM	PM

* Based on OEN study requirements regarding the privacy of Canadian data, the following masking rules are applied for the data presented here: all categories with patient numbers <10 are masked (designated with PM), all numbers ≥10 are rounded to the nearest 10, and all percentages are rounded to the nearest 5%.

† One patient had missing data on age.

‡ Data recorded at diagnosis of advanced NSCLC.

§ Data recorded at initial diagnosis of NSCLC.

‖ "De novo diagnosis" refers to patients with newly diagnosed stage III or IV NSCLC whose initial treatment was non-curative and who received a first line of SACT or BSC.

Abbreviations: BSC, best supportive care; ECOG PS, Eastern Cooperative Oncology Group performance status; IQR, interquartile range; NSCLC, non-small cell lung cancer; OEN, Oncology Evidence Network; PM, primary data masking; SACT, systemic anticancer therapy.

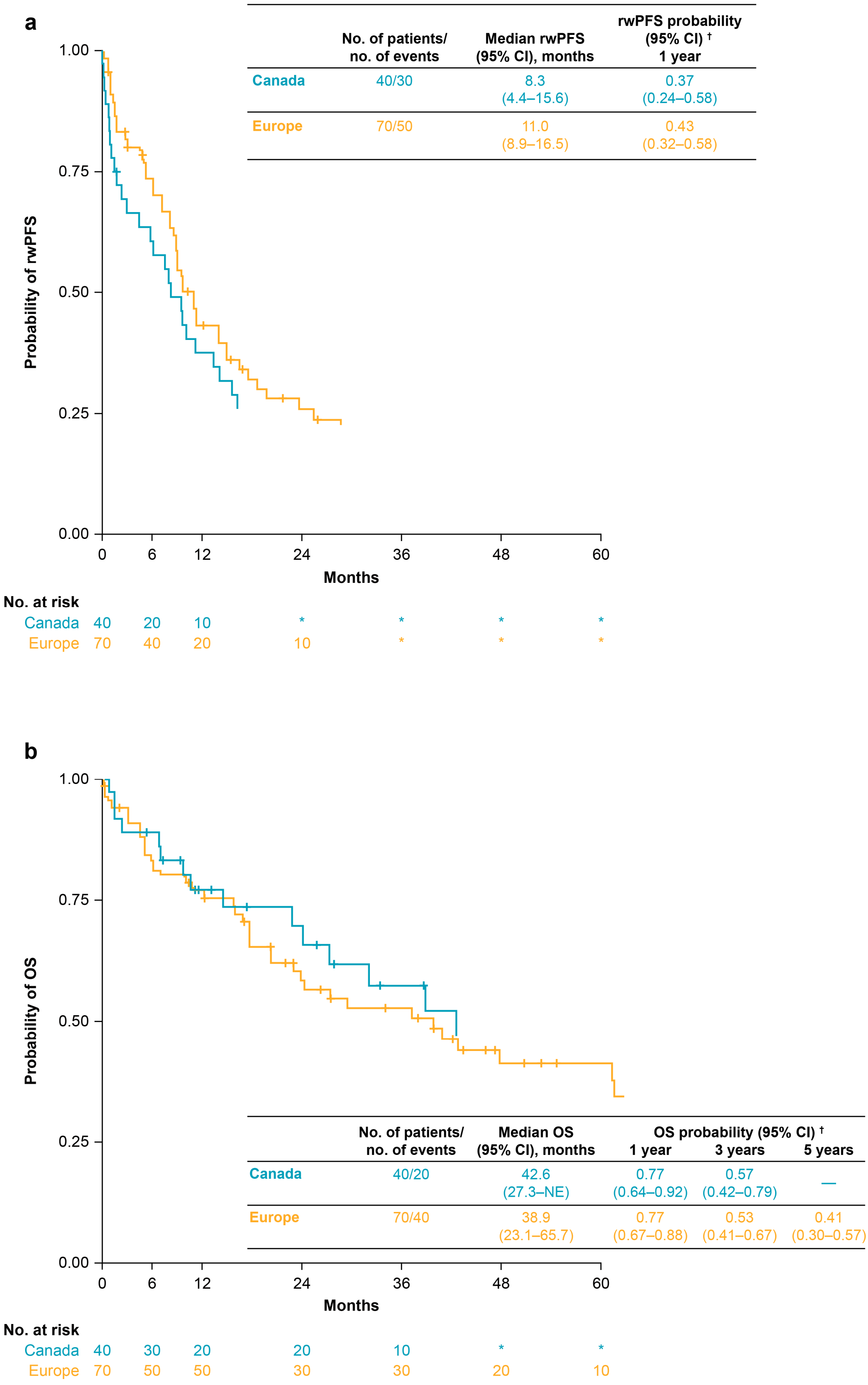

Figure S1. rwPFS (**a**) and OS (**b**) for patients receiving at least one line of SACT stratified by geographic region. Vertical tick marks indicate censored data. Due to OEN study requirements regarding the privacy of Canadian data, patient numbers between one and nine were masked (shown as an asterisk) and patient and event numbers ≥10 were rounded to the nearest 10.

† Survival probabilities are suppressed when the number of patients at risk is <10.

Abbreviations: CI, confidence interval; NE, not estimable; OEN, Oncology Evidence Network; OS, overall survival; rwPFS, real-world progression-free survival; SACT, systemic anticancer therapy.
